# Development of a Machine Learning-Based Model for Accurate Detection and Classification of Polycystic Ovary Syndrome on Pelvic Ultrasound

**DOI:** 10.7759/cureus.65134

**Published:** 2024-07-22

**Authors:** Jonathan Kermanshahchi, Akshay J Reddy, Jingbing Xu, Gagandeep K Mehrok, Fauzia Nausheen

**Affiliations:** 1 Medicine, California University of Science and Medicine, Colton, USA; 2 Internal Medicine, California Health Sciences University, Clovis, USA; 3 Medicine, California Health Sciences University, Clovis, USA; 4 Medical Education, California University of Science and Medicine, Colton, USA

**Keywords:** deep learning, endocrinology, public health, diagnostic accuracy, polycystic ovarian syndrome, pcos, ultrasound, ultrasound imaging techniques, deep neural network, artificial intelligence (ai) in medicine

## Abstract

Polycystic ovary syndrome (PCOS) is a common endocrine disorder that disrupts reproductive function and hormonal balance. It primarily affects reproductive-aged women and leads to physical, metabolic, and emotional challenges affecting the quality of life. In this study, we develop a machine learning-based model to accurately identify PCOS pelvic ultrasound images from normal pelvic ultrasound images. By leveraging 1,932 pelvic ultrasound images from the Kaggle online platform (Google LLC, Mountain View, CA), we were able to create a model that accurately detected multiple small follicles in the ovaries and an increase in ovarian volume for PCOS pelvic ultrasound images from normal pelvic ultrasound images. Our developed model demonstrated a promising performance, achieving a precision value of 82.6% and a recall value of 100%, including a sensitivity and specificity of 100% each. The value of the overall accuracy proved to be 100% and the F1 score was calculated to be 0.905. As the results garnered from our study are promising, further validation studies are necessary to generalize the model’s capabilities and incorporate other diagnostic factors of PCOS such as physical exams and lab values.

## Introduction

Polycystic ovary syndrome (PCOS) stands as a prominent endocrine heterogeneous disorder that affects 6-10% of reproductive-aged women worldwide [1]. The syndrome is often presented with chronic anovulation as well as hyperandrogenism syndromes such as acne and hirsutism [2]. In addition, metabolic disturbances such as insulin resistance and hyperinsulinemia tend to play a role in the pathogenesis of PCOS. However, there is an overlap between the normal characteristics of puberty and the presenting symptoms of PCOS. Common to PCOS is an elevated luteinizing hormone (LH) concentration, which creates menstrual abnormalities [3]. As a result, an accurate diagnosis of PCOS early in the path of disease is crucial to prevent long-term complications such as infertility, metabolic syndrome, type 2 diabetes, and increased risk of endometrial carcinoma.

Historically, the diagnosis of PCOS has relied on several factors pointing toward the disorder. Such diagnostic tools have included hormonal, metabolic, and biochemical parameters [4]. Increased body mass index plays a role in diagnosis due to the increase of the enzyme aromatase, which converts the already increased androgens circulating in the body into estrogen, which amplifies the damaged reproductive outcomes [5]. In fact, PCOS symptoms commonly improve with a 5-10% weight loss [5]. Improvement of PCOS with weight management reinforces the importance of early diagnosis for appropriate management to be initiated. Hormonal levels also aid in the correct diagnosis of PCOS. An increase in LH has been proven to correlate with PCOS and is a valuable asset in the diagnosis of the disorder [3].

Pelvic transvaginal ultrasonography has become the gold standard in imaging the presence of multiple small follicles in the ovaries and an increase in ovarian volume [6]. Ultrasound imaging is widespread and typically easy to access in a healthcare setting. However, the issue with this imaging modality is that it relies upon an interpretation of the image from a healthcare provider, which is subject to inevitable human errors as well as variability in diagnosis.

Medical imaging has witnessed a large shift in recent years with the use of deep learning and artificial intelligence (AI) technology. AI has been introduced in the field of medicine as having the potential to read different imaging modalities in health care, such as X-rays and ultrasound images. This shift brings about new possibilities for enhancing the accuracy and availability of the diagnosis of PCOS when using AI to analyze pelvic ultrasounds. Since AI has already demonstrated the potential to learn increasingly complex radiographic patterns from various pathologies, there is a promising track to a more robust and efficient detection of polycystic ovaries using pelvic ultrasound.

The primary focus of this study is to explore the capability of machine learning algorithms in enhancing the diagnosis of PCOS by creating a polycystic ovaries detection model by using pelvic ultrasound images. The implementation of AI imaging detection holds the potential to enhance the diagnostic work-up of PCOS, leading to both benefits to the patients and healthcare providers. PCOS has proven to take a toll on a patient’s quality of life. Making AI detection of polycystic ovaries a standard and reliable tool for physicians could possibly curtail inaccuracies in diagnosis. In turn, the possibility of an AI polycystic ovaries detection model using pelvic ultrasound images can lead to faster detection of polycystic ovaries, swift management initiation, and continuous monitoring through follow-up clinic visits.

## Materials and methods

**Figure 1:**
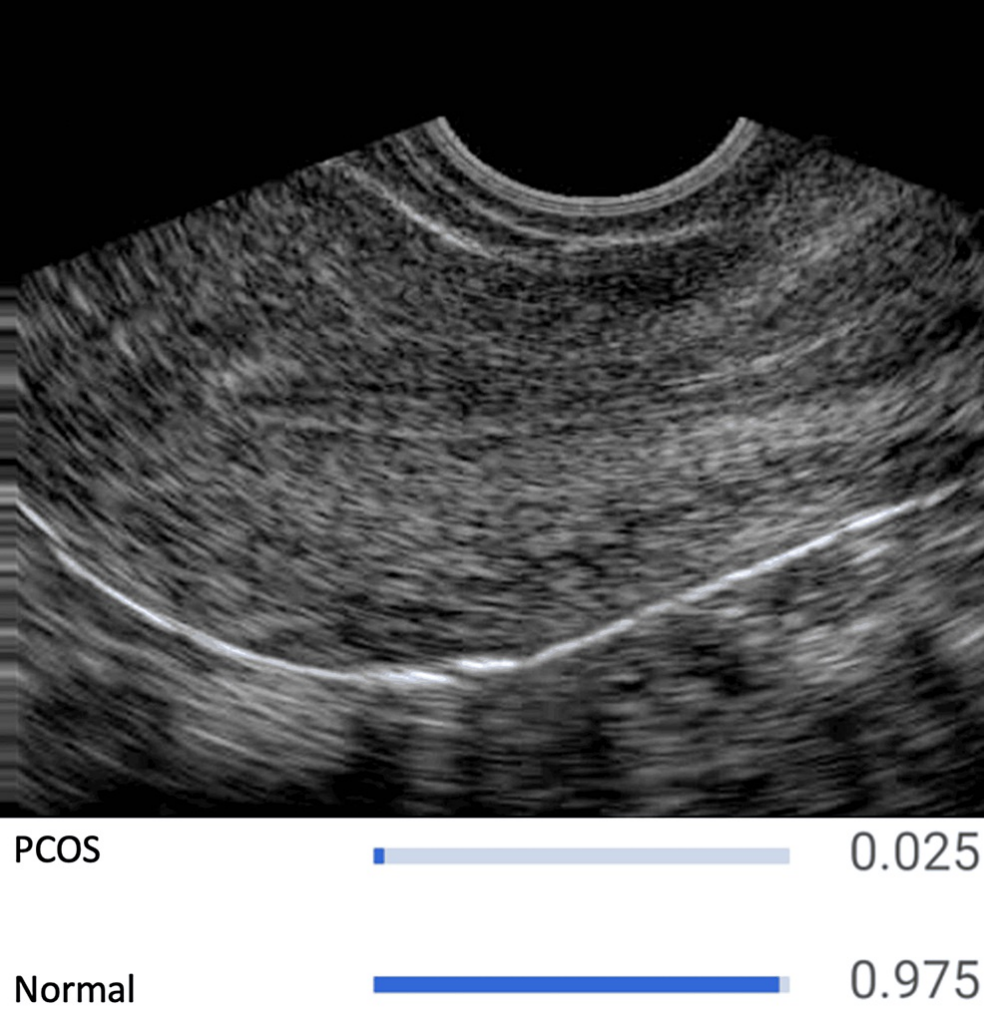
Developed CNN model detecting normal pelvic transvaginal ultrasonography image. CNN: convolutional neural network; PCOS: polycystic ovary syndrome.

**Figure 2:**
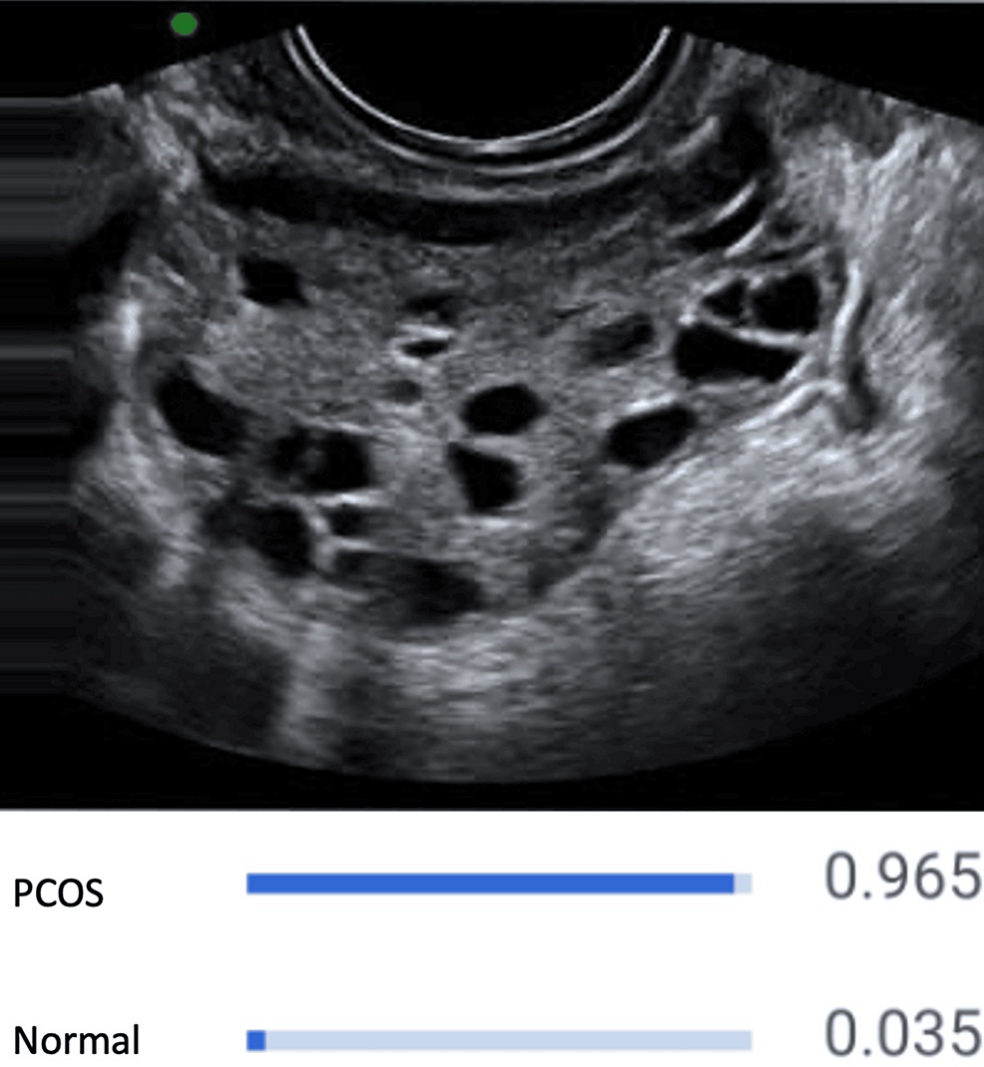
CNN model detecting PCOS on pelvic ultrasound image. CNN: convolutional neural network; PCOS: polycystic ovary syndrome.

This investigation utilized pelvic transvaginal ultrasonography images from datasets found on the Kaggle online platform (Google LLC, Mountain View, CA) [7]. The dataset used contains over 1,932 pelvic ultrasound images, including 1,145 normal pelvic ultrasound images (Figure 1) and 787 polycystic ovaries-positive ultrasound images (Figure 2). Before processing, every image was meticulously inspected for proper quality.

Deep learning algorithms, particularly convolutional neural networks (CNNs) were used in this study and have demonstrated outstanding capabilities in object detection tasks. CNNs employ cascades of filters to acquire specific features from images, such as in the images presented in the dataset used for this study. This process empowers the system to discern patterns found in the images. Google’s collaboration platform has been utilized for model development for this investigation.

All assignments in the study were randomized and three categories of the dataset were utilized to structure the learning model. The first category includes training, followed by validation, and finished by testing. The use of training the model (80% of images) served the detection system by enhancing the model’s capabilities. To avoid overfitting, the validation set was utilized (10% of images). The testing set served to evaluate the success of the model (10% of images). The performance of the deep learning algorithms was evaluated by its success in correctly identifying positive PCOS pelvic ultrasound images (post-diagnosis from a trained physician) in contrast to normal pelvic ultrasound images.

To quantify the performance of the model, accuracy, precision, recall, and confusion matrix were calculated. The software tools used for this model included Python (Python Software Foundation, Wilmington, DE), TensorFlow (Google, Mountain View, CA), and PyTorch (Meta AI, Astor Place, NY), as well as Google’s collaboration platform was used.

## Results

**Figure 3:**
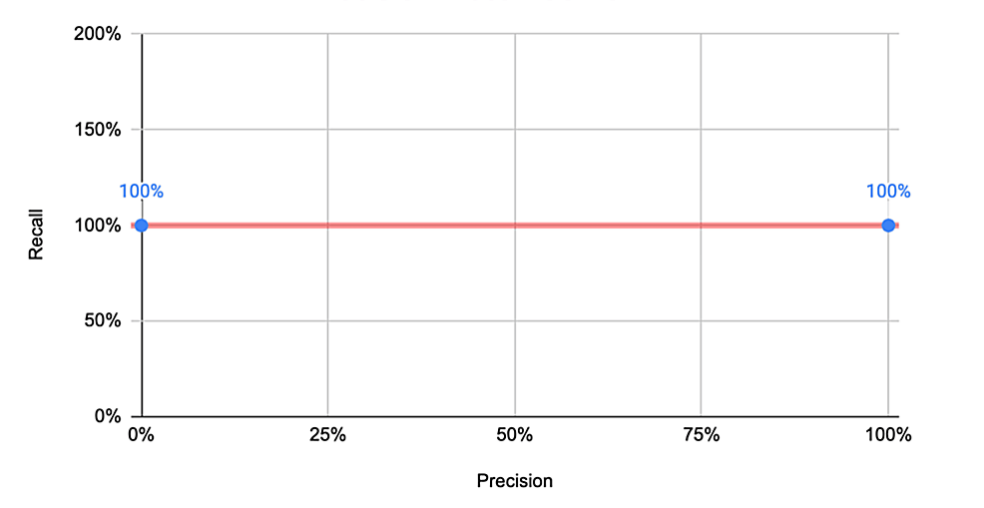
AUC graph for polycystic ovaries detection model including recall and precision. AUC: area under the curve.

An encouraging and exciting performance was exhibited from the polycystic ovaries detection model. The model remarkably reached an area under the curve (AUC) of 1, which demonstrates the model’s success at discriminating between normal pelvic ultrasounds and polycystic ovaries-positive ultrasounds. Interestingly, precision was found to be 82.6% while the recall value lay at 100%. This promising performance is displayed in Figure 3.

One problem that may be present in this study from an elevated recall value would be the overfitting of the model. The ideal outcome for the model used would be for the model to only engage in deep learning to be able to distinguish the two types of images. However, overfitting may lead to the machine learning algorithm memorizing the data rather than learning from patterns. This may mean that the model may not be able to perform active and deep learning because it may be memorizing instead of learning.

**Figure 4:**
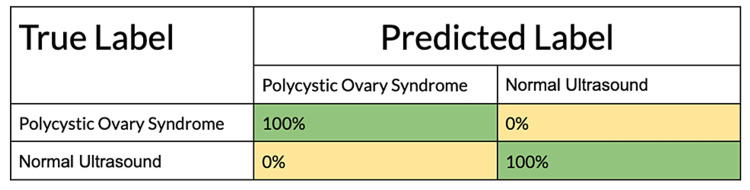
Confusion matrix.

To completely investigate the model’s efficacy, more statistical analysis was done. This analysis sought to evaluate the sensitivity, specificity, and overall accuracy of the model. This was found from Figure 4, the confusion matrix.

Impressively, the sensitivity (true positive rate) was calculated to be 100%. The interpretation of this is that the model correctly identified 100% of the polycystic ovaries-positive pelvic ultrasound images when compared to the normal ovary images. In addition, the true specificity (true negative rate) was found to also be at 100%. This means that when the model confronts a pelvic ultrasound image, the deep learning machine proves to be 100% efficient in detecting and labeling normal pelvic ultrasound images. It is important to also calculate the accuracy of the deep learning machine. This is an overall statistic that demonstrates the proportion of polycystic ovaries-positive ultrasound images and normal pelvic ultrasound images that were classified by the model correctly. The value of the overall accuracy for this model was found to be 100%, which highlights the promising success of deep learning algorithms in differentiating between normal pelvic ultrasounds and pelvic ultrasounds that are positive for polycystic ovaries. The F1 score was found to be 0.905.

## Discussion

Our study looked into the potential of a machine learning algorithm to discern a normal pelvic transvaginal ultrasound image from a pelvic transvaginal ultrasound image with polycystic ovaries. PCOS is an imbalance of sex hormones in the body, especially the excess of androgens and insulin characterizes PCOS [6]. These imbalances play a role in disrupting the normal function of ovaries. Hormonal imbalance can lead to menstrual cycle abnormalities, which decreases fertility in patients with PCOS [6]. Along with patient history and physical exam signs, pelvic ultrasound imaging plays a crucial role in diagnosing PCOS, which shows multiple cyst cavities in the ovaries. Early detection of polycystic ovaries through pelvic ultrasound imaging is vital to improve quality of life by implementing interventions for patients who suffer from reproductive disorders [8]. Therefore, deep learning detection models, such as ours, have the potential to greatly improve the care for those who suffer from PCOS.

The ability to accurately detect polycystic ovaries early in their manifestation is crucial to initiating effective management for the improvement of a patient’s quality of life. The diagnosis of PCOS means women with previously unexplained and troublesome symptoms will undergo a more efficient diagnostic process to receive a diagnosis of PCOS, which would prompt treatment, including lifestyle change, so PCOS symptoms could be improved [9]. Pelvic ultrasound images are found to be widespread around the globe, and having a uniform and reliable model to differentiate between polycystic ovaries-positive cases and normal pelvic ultrasounds can greatly enhance the care of this pathology. The combination of the widespread availability of ultrasound makes machine learning technology the ideal system for analyzing detection models around the world.

The overall accuracy of the model is found to be at an outstanding 100%, which demonstrates the promising use of this model for the future of PCOS care. PCOS affects many aspects of the quality of life of those who suffer from it. This includes infertility, excess body hair, and acne or oily skin, which all contribute to a net negative effect on a patient’s emotional well-being [10]. The utilization of polycystic ovary detection models can serve as a considerable ally in making informed clinical decisions regarding the hormonal management of the patient, which can therefore lead to an improvement in patient outcomes.

As the results of the model are promising, it is important to not overlook the limitations of this study. To begin with, the size of the dataset used to extract pelvic ultrasound images from Kaggle.com and the unreliability of the imaging quality of the images could have influenced the results from the model studied. Also, the results of AI in detecting PCOS are influenced by the deep learning algorithm used for the study. Variations in different deep learning algorithms may influence the performance of the detection model.

The sole factor in predicting PCOS in this model relies on pelvic ultrasound images. There are more diagnostic criteria in diagnosing PCOS, which include a physical inspection and exam as well as lab values. Clinical/biochemical hyperandrogenism and anti-Müllerian hormones also play a role in the diagnosis of PCOS. Future studies should look into including more diagnostic tests to create a full picture of the deep learning algorithm’s effect on PCOS.

## Conclusions

This research showcases the capabilities of deep learning algorithms in creating an accurate model of detecting polycystic ovaries from pelvic ultrasound images. The polycystic ovaries detection model proved to have an accuracy of 100%, and other statistical measurements testing the model demonstrated outstanding results. To increase the generalization of the model and the ability for the model to play an effective role in working with healthcare professionals, additional research with different datasets and usage of other criteria of PCOS diagnosis is essential. As a result of implementing machine learning and deep learning algorithms in the detection of polycystic ovaries, an increase in diagnostic accuracy and therefore an earlier detection of polycystic ovaries has the potential to lead to an earlier initiation of treatment and better patient outcomes.
